# Examining Monetary Valuation Methods to Analyze Residents' Social Value From Hosting a Publicly-Funded Major Sport Event

**DOI:** 10.3389/fspor.2022.823191

**Published:** 2022-03-23

**Authors:** Jordan T. Bakhsh, Marijke Taks, Milena M. Parent

**Affiliations:** School of Human Kinetics, Faculty of Health Sciences, University of Ottawa, Ottawa, ON, Canada

**Keywords:** outcomes, legacy, mixed methods, social return on investment, sport event, social value

## Abstract

Measuring residents' social value from hosting major sport events has become a popular practitioner and researcher focus. However, researchers have used a plethora of monetary valuation methods to measure social value on an equally diverse set of events. Rather than being applied to major sport events, the use of these methods in sport research has been limited to smaller events, programs, or facilities. Consequently, investigating monetary valuation methods for major sport events is necessary to inform practitioners and researchers of these types of events as to which tool(s) to use. Thus, the purpose of this study was to investigate various monetary valuation methods to determine which method(s) is(are) best to examine residents' social value in a post-event context and test the selected method(s) for the 2010 Olympic Winter Games in Vancouver, Canada. After reviewing monetary valuation methods found in the sport management literature, two methods were deemed suitable avenues to pursue: the reverse contingent valuation method and the opportunity cost approach. This study employed an exploratory sequential mixed methods design to derive a conceptual and empirical analysis. Interviews were conducted with 14 Vancouver residents and supplemented with document analysis; as well, 525 Vancouver residents completed a self-administered online survey. Findings highlighted the importance of using both the reverse contingent valuation method and opportunity cost approach given their complementary nature. The reverse contingent valuation method allowed residents to select how much they valued their experience. This individual or micro-economic perspective is a necessary prerequisite for residents to adequately determine their value of hosting in relation to other options (e.g., building hospitals, having professional sport teams) when applying the opportunity cost approach, which asks residents to reflect at societal or macro-economic level. This synergistic approach demonstrates the importance of addressing both perspectives: the micro (i.e., individual exchange) and the macro (i.e., event exchange) aspect. In doing so, this approach offers researchers and practitioners avenues forward to examine the social value of publicly-funded major sport events exclusively through a direct, an indirect, and a synergistic method to advance the examination of major sport events' social value.

## Introduction

Gone are the days of organizations justifying the value of their activities solely through financial proxies (Norman and MacDonald, 2004). As the desires of consumers and investors have evolved, so too has the need for organizations to show how their organizational activities create social value (Maldonado and Corbey, 2016). This focus on social value is no different with sport events (e.g., Misener and Schulenkorf, 2016), as sport is believed to have an inherent social value (Slack, 1998) that should be measured but is a challenging endeavor (Sam and Rongland, 2018). Nevertheless, this inherent belief has propelled sport stakeholders to claim that hosting major sport events, like the Olympic Games, can create social value for communities that justify their public economic investment (Solberg et al., 2016; Doyle et al., 2021).

Social value is the value that individuals attribute to experiences (Schumpeter, 1909) and includes a combination of market and non-market goods (Orlowski and Wicker, 2019). As important as social value is, equally important is how social value is produced and measured (O'Flynn, 2007). To appropriately measure social value, one's experiential changes (i.e., outcomes) must be measured in the same unit as their inputs (King, 2014). Thus, in the case of publicly-funded major sport events, taxpayers' money (i.e., input) is used to stage the event; hence residents' social outcomes should be monetized. While market goods and services (i.e., prices at which a good/service is being sold/bought; Orlowski and Wicker, 2019) are available in monetary units, non-market goods/services, like consumer surplus (Brynjolfsson et al., 2003) and public good value (Brookshire and Coursey, 1987), have no price, making it more difficult to measure. This challenge often excludes these important non-tangible outcomes from social value analyses (Maldonado and Corbey, 2016; Keane et al., 2019; Gosselin et al., 2020).

A popular process to monetize these non-market values is through using a monetary valuation method: the practice of converting something intangible (e.g., experience) into a monetary unit (Walker and Mondello, 2007). Done through a monetary valuation method (e.g., contingent valuation method; Funahashi et al., 2020), a common unit of measure (i.e., money) is created, which can then facilitate objective evidence-based decision-making (Clark and Oswald, 2002). However, based on our current knowledge, a series of gaps exist when applying monetary valuation methods to understand residents' social value from hosting major sport events.

First, scholars examining the monetary value of major sport events have exclusively focused on event microcosms like event volunteers (e.g., Downward and Dawson, 2016), sport outcomes (e.g., Mutter and Pawlowski, 2014; Humphreys et al., 2018), recreation facilities (e.g., Davies et al., 2021), and recreation programs (e.g., Davies et al., 2019). Although these were good initial steps, a monetary valuation of residents' major sport event social perspectives (i.e., experiences, perceptions, and insights) is noticeably missing (Keane et al., 2019; Gosselin et al., 2020). Although researchers and practitioners can certainly learn from these initial studies, we cannot amalgamate these microcosms to form an understanding of the event's overall social value. Doing so can create measuring challenges since multiple event aspects contribute to social value, and it is difficult for researchers and respondents to compartmentalize these aspects and their specific social value when examining overall social value (Lingane and Olsen, 2004).

Second, although a variety of monetary valuation methods have been used to address sport event queries (Orlowski and Wicker, 2019), major sport events include a variety of contextual characteristics which differ between events (e.g., culture, public perception, politics; Parent and Ruetsch, 2021). These varying characteristics challenge our ability to compare event projects, as a comparison must occur on the same target market (Maldonado and Corbey, 2016). Since scholars have often exclusively used one method (e.g., Boronczyk and Zarins, 2020; Funahashi et al., 2020), a rich landscape of varying methods applied to different event contexts have been created. Consequently, as sport management scholars, although many tools may be used to measure the overall social value from hosting a major sport event, we do not know which tool(s) is(are) best for examining residents' social value from hosting a major sport event.

This lack of a major sport event's monetary valuation and the lack of any cross-method comparison amongst sport event studies leaves researchers and practitioners at a standstill, uncertain as to which method(s) is(are) best to address these stakeholder claims and examine the social value from hosting a major sport event (Orlowski and Wicker, 2019; Davies et al., 2021). Consequently, a comparative examination should occur to determine which monetary valuation method(s) is(are) best for examining the social value of a major sport event. Reviewing monetary valuation methods and empirically testing various monetary valuation methods will offer researchers the opportunity to better understand and evaluate social value from major sport events. It presents practitioners and residents with more transparency and assists in making more informed decisions around hosting publicly-funded major sport events. Therefore, the purpose of this study was to investigate various monetary valuation methods to determine which method(s) is(are) best to examine residents' social value in a post-event context and test the selected method(s) for the 2010 OWG.

## Literature Review

This section provides a broad overview of monetary valuation methods. By reviewing the various types of monetary valuation methods used within sport management scholarship, this section offers insights into which methods can be applied to analyze residents' monetary valuation from hosting the 2010 OWG based on specific selection criteria.

### Monetary Valuation

Individuals use monetary valuation with daily activities, from store transactions (i.e., product for currency) to allocating time and resources (e.g., time spent working out vs. time spent working; Downward et al., 2009). Rooted in welfare economic theory, monetary valuation defines value as the trade-off individuals are willing to make between two or more goods/services (Segerson, 2017). This notion is connected to the concept of utility, which is concerned with one's choices, decisions, and value (Fishburn, 1968). The driving principle of utility is to analyze one's value and choice based on their actions and preferences (Rothburn, 1956). Within economic theory, there are two dominant perspectives: *micro* and *macro*. The micro perspective focuses on the decisions, values, and exchanges of individuals, or in the case of major sport events, event stakeholders (e.g., residents; Wicker et al., 2012). In contrast, the macro perspective focuses on the decisions, values, and exchanges of countries, governments, or in the case of major sport events, the event itself (Wicker et al., 2012). Scholars can measure micro and macro perspectives of social value through market or non-market prices and appropriate hypothetical scenarios (Orlowski and Wicker, 2019).

Scholars have applied monetary valuation methods to measure one's social utility or social value (Orlowski and Wicker, 2019). Given the universal value monetary units hold for individuals and organizations (Lapavitsas, 2005), one may think to measure the monetary value of a good/service by (simply) using market prices. For instance, when an individual purchases a fitness membership, the individual's intangible value is reflected by the cost of that membership. However, this does not comprehensively reflect an individuals' actual value of their exchange, as both monetary and non-monetary units (i.e., market prices and non-market prices; Becker, 1965) can be involved in the exchange, which can result in notions of consumer surplus (Brynjolfsson et al., 2003). Moreover, when purchasing and using a fitness membership, additional complexities like the individual's experience, nature of the market, relationships fostered in this space, opportunities foregone (e.g., time spent working; Becker, 1965), and much more are not considered within these valuations (Orlowski and Wicker, 2019). Thus, a key challenge of traditional valuations for sport contexts, like major sport events, is individuals do not need to consume the good/event to receive utility from it (Carson, 2000).

Orlowski and Wicker (2019) critically assessed 113 articles which provided a comprehensive overview of multiple valuation approaches. From this analysis, the authors identified 12 valuation methods which can be categorized into three types: (1) revealed preferences; (2) stated preferences; and (3) hybrid methods. To serve the purpose of this study, namely determining residents' social value from hosting an event many years after it occurred, a monetary valuation method needs to meet various criteria. First, the method must be able to conduct a post-event valuation. Second, since social experiences do not have a market price (i.e., are not a product for purchase: Orlowski and Wicker, 2019) the method cannot rely on market values. Finally, since social experiences are a holistic concept and not necessarily one niche aspect (Lingane and Olsen, 2004), the method must be able to value the entire experience, rather than multiple individual aspects combined. In the following section, the applicability of the valuation methods presented by Orlowski and Wicker (2019) will be evaluated based on the three identified selection criteria: (1) ability to conduct a post-event evaluation; (2) not requiring market values; and (3) providing a monetary value of social experiences.

#### Revealed Preferences

The central tenet of monetary valuation revealed preference methods is that individuals reveal their true preferences through action (Orlowski and Wicker, 2019). Therefore, data on individuals' behavior/actions are collected and analyzed to understand their preferences. Sport management researchers have used various types of revealed preference methods, including *compensating variation approach* (e.g., Downward and Dawson, 2016), *hedonic pricing* (e.g., Feng and Humphreys, 2018), *opportunity cost* (e.g., Salamon et al., 2011), *replacement cost approach* (e.g., Vos et al., 2012), and *travel cost method* (e.g., Melstrom et al., 2017).

*Hedonic pricing, replacement cost approach*, and *travel cost method* rely on the direct association of use-values or market pricing (e.g., cost of a plane ticket or hotel accommodations; Orlowski and Wicker, 2019). This aspect challenges their applicability for this study's major sport event valuation because not all aspects of such exchange can be connected to market prices (e.g., psychic income; Oja et al., 2018). For instance, *hedonic pricing* can only assign monetary values to non-market goods directly associated with use-values (e.g., real estate; Orlowski and Wicker, 2019). This aspect challenges the ability for non-use values (e.g., social value) to be considered, which are inherent to residents of a major sport event—regardless of their chosen consumption of the event, they are directly and indirectly affected by its presence (Karadakis and Kaplanidou, 2012; Bakhsh et al., 2018). Consequently, monetary valuation methods that rely on such market prices do not meet the criteria needed to be included in this study. Despite the challenges present amongst these revealed preference methods, the *compensating variation approach* and *opportunity cost approach* are not subject to these difficulties and are thus potential methods to examine the value of a major sport event.

##### Compensating Variation Approach

This approach estimates the amount of income an individual is willing to forego to consume a greater amount of a certain good/service while maintaining their utility level (Powdthavee, 2008). In other words, how much one is willing to pay to increase one's experiential aspect while maintaining their utility (Downward and Dawson, 2016; Orlowski and Wicker, 2018). For example, Downward and Dawson (2016) tested this in a study on 16,627 individuals' sport participation choices and well-being. Data were first determined by individuals behavior and experience scores and self-reported subjective wellbeing or quality of life type metrics (e.g., Downward and Rasciute, 2011). Then, individuals were asked to evaluate the change in their subjective wellbeing from participation experiences. These changes were then expressed in monetary terms and various value options were provided.

Consequently, when monetary values are not readily available (e.g., hypothetical scenarios), applying this method can lead to hypothetical decisions that diminish the tangible goal of applying monetary valuation methods. Often used in relation to positive sport participation outcomes (e.g., Downward and Dawson, 2016; Orlowski and Wicker, 2018), there is a lack of negative outcomes monetized by sport management scholars (Orlowski and Wicker, 2019). However, beyond sport management scholarship, negative outcomes like noise have also been studied using a compensating variation approach (e.g., Van Praag and Baarsma, 2005). For instance, Van Praag and Baarsma (2005) investigated the monetary value of noise damage on housing costs caused by aircraft noise of residents living beside an airport.

A conceptual challenge with the *compensating variation approach* is the assumption that individuals can compartmentalize their experiential aspects (e.g., social outcomes) when, in reality, social experiential aspects (e.g., social capital, psychic income; Oja et al., 2018) are challenging to untangle and to evaluate objectively (Taks et al., 2020). Moreover, untangling these experiential event aspects would not allow for the macro monetary valuation of a major sport event sought in this study. Instead, it offers insight into the value of event microcosms which have been the focus of major sport event monetary valuations to date (e.g., Downward and Dawson, 2016; Humphreys et al., 2018). Therefore, obtaining measures for a major sport event with this method would be riffled with issues, as aspects of social experiences cannot easily be pulled apart like bricks of a building—the experience is inclusive to all aspects, and the entire experience itself must be considered (Lingane and Olsen, 2004). Consequently, although *compensating variation approach* meets the first two criteria required for this study (i.e., post-event and does not rely on market values), it does not evaluate an entire social experience and does not meet the final criteria needed to be applied in this study.

##### Opportunity Cost Approach

This approach examines the cost of the best but not chosen alternative option, to evaluate the value of a particular course of action (i.e., opportunity cost; Késenne, 2012). In principle, this method can be used in any exchange where an alternative option can be posed. For example, within sport management research, scholars have used this method to consider the value of volunteer work, with the opportunity cost being the salary/income foregone in exchange for the individual's voluntary service (e.g., Salamon et al., 2011; Orlowski and Wicker, 2015). Although applicable in principle, two challenges are present when applying this method. First, the most common concerns focus on potential variations within the alternative option. For instance, when using time spent working as the alternative to time spent volunteering, the same voluntary act can yield different monetary values depending on the individual being analyzed (e.g., lawyer or student). Although this concern holds merit, alternatives (e.g., providing individuals with a hypothetical set income) pose challenges to value authenticity, as individuals in a hypothetical situation are inherently making hypothetical decisions that diminish the tangible goal of applying monetary valuation methods. Second, the ability to confirm a monetary value for the best alternative option poses a challenge when applying this method. Depending on the scenario, the best alternative option of the non-use value may be another non-use value, challenging the tangibility of the findings. Despite these (inherent) limitations to applying the *opportunity cost approach* to sport contexts, the principles of this method can provide insightful understandings of an individual's value and relative value (Orlowski and Wicker, 2019). For example, from evaluating the foregone option(s) of residents' public funding, in relation to other use and non-use values, a greater understanding of the relative value of a major sport event can be learned. Consequently, beyond evaluating values of the best alternative option forgone, the notion of preferences cannot be denied and is an important aspect to consider with this macro valuation. Thus, the *opportunity cost approach* meets the three criteria needed to be applied in this study and will be retained to analyzing residents' monetary value of hosting a major sport event at a macro level.

#### Stated Preferences

Unlike revealed preference methods, stated preference methods address the monetary valuation of future or hypothetical behaviors (Orlowski and Wicker, 2019). In this case, data were collected by presenting individuals with a hypothetical future scenario and recording their willingness to pay for this scenario (e.g., Johnson and Whitehead, 2000). Sport management scholars have used various types of stated preference methods, including *choice modeling* (e.g., Bertram et al., 2017), *contingent behavior method* (e.g., Whitehead et al., 2013), and *contingent valuation method* (e.g., Boronczyk and Zarins, 2021).

Although these methods have been applied in sport-related studies because of their hypothetical/future scenario nature, they are limited to evaluating an event's potential or expected monetary value rather than offering insights on a post-event value. This hypothetical nature is problematic for the present study's context, as each method is challenged to offer a value of a major sport event *post-hoc*. However, this does not discount the insight they can provide for other contexts, as understanding the potential value of a major sport event can be an insightful tool to garner resident support and effectively plan the allocation of public funding to host. Nonetheless, the hypothetical bias present in these methods (Orlowski and Wicker, 2019) challenges their applicability for analyzing residents' post-event monetary value of hosting a major sport event. Despite the ex-ante and *post-hoc* challenges present in stated preference methods, an innovative approach to contingent valuation, *reverse contingent valuation* (e.g., Humphreys et al., 2018), offers a stated preference method that allows *post-hoc* analysis.

##### Reverse Contingent Valuation Method

This method follows the same principles as contingent valuation method; however, while those methods are conducted pre-event and do not meet the criteria for this study, reverse contingent valuation method determines individuals' preferences post-experience rather than pre-experience (Humphreys et al., 2018). *Reverse contingent valuation method* is a survey-based method used to directly elicit individuals' willingness to pay for non-market goods/services (Carson, 2000) after the fact. A value expressed through this method blends social and economic attributes from experience consumption (Ciriacy-Wantrup, 1947). Using this method provides a relatively high level of flexibility with (a) timeframe (e.g., one-time or aggregate evaluations); (b) payment vehicle (e.g., voluntary donations, annual taxes); and (c) response formats (e.g., dichotomous choice, open question; Frick and Wicker, 2018). A *reverse contingent valuation method* allows individuals to determine their event value after having the opportunity to experience/host the event, which makes it advantageous for researchers looking to directly examine residents' investment in and outcomes from major sport events at a macro level. Thus, the *reverse contingent valuation method* meets the three criteria needed to be applied in this study and will be retained to analyzing residents' monetary value of hosting a major sport event at a macro level.

#### Hybrid Methods

Hybrid methods combine revealed and stated preference monetary valuation methods. The combination of two (or more) methods is used to combat select weaknesses of one method with select strengths of another approach (Whitehead et al., 2013). Within sport management research, three types of hybrid methods have been used, all in combination with the *travel cost method*: (1) *contingent valuation method* (e.g., Vial and Barget, 2021); (2) *contingent behavior method* (e.g., Wicker et al., 2017); and (3) *choice modeling* (e.g., Paulrud and Laitila, 2004; Orlowski and Wicker, 2019). However, since the individual methods which comprise present hybrid methods did not meet the selection criteria needed for the present study (i.e., *travel cost method, contingent valuation method, contingent behavior method, choice modeling*), their combination would also not meet the selection criteria needed. Thus, none of these three hybrid methods can be applied in this study to analyze residents' monetary value of hosting a major sport event at a macro level post-event. Table 1 summarizes the selection criteria of the revealed preferences, stated preferences, and hybrid monetary valuation methods investigated.

**Table 1:** Review of monetary valuation methods.

**Method**	**Selection criterion**		
	**1: Ability to conduct post-event evaluation (yes/no)**	**2: Does not require market values (yes/no)**	**3: Can provide monetary value of social experiences (yes/no)**
**Revealed preferences**			
Hedonic pricing	Yes	No	No
Replacement cost approach	Yes	No	No
Travel cost method	Yes	No	No
Compensating variation approach	Yes	Yes	No
**Opportunity cost approach**	**Yes**	**Yes**	**Yes**
**Stated preferences**			
Choice modeling	No	Yes	Yes
Contingent behavior model	No	Yes	Yes
Contingent valuation method	No	Yes	Yes
**Reverse contingent valuation method**	**Yes**	**Yes**	**Yes**
**Hybrid methods**			
Travel cost method + Contingent valuation method	No	No	Yes
Travel cost method + Contingent behavior model	No	No	Yes
Travel cost method + Choice modeling	No	No	Yes

*The bolded values indicate the methods which meet the selection criteria and can be used in the study*.

Although these hybrid methods are not applicable for this study, the idea of a hybrid method (i.e., combining monetary methods) could still be used to effectively measure residents' social value from hosting a major sport event. To do so, combining the methods which meet the selection criteria (i.e., *opportunity cost approach* and *reverse contingent valuation method*) could prove to be an adequate tool for researchers and practitioners. The combination of the *reverse contingent valuation method* and *opportunity cost approach* has not been used by scholars to date, but the conceptual underpinnings can be linked to the complementary micro and macro economic theory perspectives. On the one hand, the micro perspective is offered by the *reverse contingent valuation method*, as this method allows individuals to make their individual (i.e., micro) decisions and value regarding their taxpayer contributions within this socio-economic exchange. On the other hand, the macro perspective is offered by the *opportunity cost approach*, as this method allows individuals to evaluate the choice to use the overall public funding to host the 2010 OWG in relation to foregone alternatives. By concurrently examining both perspectives (i.e., micro then macro), researchers and practitioners can understand the importance of each method and their (potential) complimentary nature when examining residents' social value from hosting a major sport event can be understood. Although in theory, micro and macro perspectives are complimentary, the following empirical analysis may reveal if one or the other is more important to determine social value of events *post-hoc*, or whether a combination of both is more beneficial.

Thus, conducting a study that applies a *reverse contingent valuation method* and *opportunity cost approach* to examine residents' social value from hosting a major sport event would provide valuable information toward the applicability of each method, their (potential) complementary results, and their ability to be incorporated (or not) into a hybrid method. In addition, evidence-based justifications could be made toward developing a method to more effectively examine residents' monetized social value from hosting a major sport event.

## Method

### Research Context

This study's major sport event context was the 2010 OWG hosted in Vancouver and Whistler, Canada. Following a positive public referendum, where City of Vancouver residents voted in favor of hosting (i.e., 64%; VANOC, 2009), the City of Vancouver and the Resort Municipality of Whistler were successfully awarded the hosting rights in 2003 to organize the 2010 OWG (IOC, 2014, 2020). To operationalize the hosting of the 2010 OWG, a combination of IOC contributions, public funds [i.e., taxpayer contributions via government(s)], sponsorship, and ticketing revenues were used (Van Wynsberghe, 2016).

As a result, residents became engaged in a socio-economic exchange where residents provided public funding in the form of tax dollars to host the 2010 OWG in exchange for social experiences (i.e., intangible outcomes).^1^ Now, more than 11 years post-event, residents are afforded the ability to evaluate the event based on actual lived experiences rather than perceptions and reflect on its social value *post-hoc*. The combination of these key elements—(1) positive public referendum; (2) use of public funding; and (3) post-event experiential evaluations—makes examining residents' social value from hosting the 2010 OWG an ideal case to identify and compare monetary valuation methods for the application of major sport events.

### Mixed Methods Design

We applied an exploratory sequential mixed methods design to compare the two identified monetary valuation methods (i.e., *opportunity cost approach* and *reverse contingent valuation method*) (Creswell et al., 2003). This exploratory sequential design links a qualitative phase to a subsequent quantitative phase, prioritizing data given to the qualitative phase, which informs the quantitative phase (Creswell et al., 2003). Therefore, this design is advantageous when testing new elements of theory and building or testing a new instrument/tool, as is the case in the present study (Morgan, 1998; Creswell, 2010).

According to the seminal mixed methods scholars, Tashakkori and Creswell (2007), four criteria must be met to effectively conduct mixed methods research: (1) *justification*; (2) *mixed method type*; (3) *distinct results*; and (4) *mixing*. Consequently, the following steps were taken. First, *justification*, to best compare monetary valuation methods, we first explored what inputs and outcomes were experienced by the transaction members and what options were foregone because of funding the major sport event. This knowledge was necessary to accurately depict willingness-to-pay metrics and opportunity cost in the quantitative phase. While some of these foregone options, inputs, and outcomes can be understood through document analysis (e.g., residents provided public funding for the event), there is a need to explore residents' perspectives of the event to inform how the monetary valuation methods can best be presented and disseminated in the next phase. Second, *mixed method type*, we applied an exploratory sequential mixed methods design which included semi-structured interviews (i.e., qualitative instrument) and a self-administered questionnaire (i.e., quantitative instrument). Finally, aligned with Tashakkori and Creswell's (2007) third and fourth criteria (i.e., *distinct results* and *mixing*), the following sections present the qualitative and quantitative data methods, results, and discussion, with the overall mixed methods discussion presented thereafter.

## Qualitative Method

### Qualitative Data Collection

Upon receiving ethical approval from the university's research ethics board (#XXX), data were collected from October 2020 to November 2020 through semi-structured interviews (hereafter referred to as interviews) and documents (e.g., financial documents and bid reports). Interviews were an appropriate data collection method as this study sought to understand residents' perspectives about the 2010 OWG and gauge the social value of their experience and potential foregone opportunities from hosting the major sport event (Smith and Sparkes, 2016). In addition, documents were collected as a source of triangulation, thereby increasing the trustworthiness of the research findings (Edwards and Skinner, 2009; Burke, 2016).

Through a purposeful sample and snowball sampling strategy (Edwards and Skinner, 2009), we conducted 14 interviews with residents, as no new element arose during the last couple of interviews and theoretical saturation was reached (Rowlands et al., 2016). All residents were 18 years of age or older during the event's 2003 referendum and confirmed living in the Metro Vancouver Regional District from the referendum to the interview (i.e., 17 years). This ensured all participants were of legal voting age during the referendum and could reflect on their experiences as a host resident of the 2010 OWG. Interviews ranged from 37 to 93 min in length and were all conducted via Zoom. The interview guide for these resident interviews included questions about event legacies, perceptions, experiences, and opportunities (potentially) foregone from hosting. The interview guide asked questions pertaining to event legacies, experiences, and social value from hosting the 2010 OWG. Questions were built from previous sport management scholarship which has examined residents' experiences from hosting major sport events (e.g., Bakhsh et al., 2018; Oja et al., 2018; De Rycke et al., 2019). Refer to Appendix A for the interview guide.

As a means of corroborating the findings from the semi-structured interviews (Yin, 2018), 2010 OWG archival records and documentation were used for data source triangulation (Lincoln and Guba, 1985). Specifically, the first author collected three formal documents through publicly available sources on the 2010 OWG. They included the event's formal Bid Report (VANOC, 2009, p. 54), Consolidated Financial Statements (VANOC, 2010, p. 28), and the Review of Games Estimates audit by the province of British Columbia (BC Auditor, 2002, p. 76). This document analysis was strategically executed to provide an in-depth understanding of the event's outreach, goals, use of public funding from the event's perspective, and confirmed that Vancouver residents contributed on average an estimated $75–$175 each year for 7 years prior to hosting the event, in present value, per taxpaying individual. This calculation was determined by taking the overall City of Vancouver public funding contribution and dividing it by the number of residents during the Games.

### Qualitative Data Analysis

Interviews were audio-recorded, transcribed verbatim, and sent back to participants for member checking to ensure the transcripts accurately represented their experiences (Burke, 2016). Once participants approved their transcript, data were uploaded to the qualitative data analysis software NVivo 11 Plus and analyzed thematically following Braun and Clark's (2006) six steps: (1) *familiarization with data*; (2) *generating initial codes*, including deductive and inductive; (3) *searching for themes*; (4) *reviewing themes (refinement)*; (5) *defining and naming themes*, where the researchers develop descriptive and analytical interpretations of the data; and (6) *producing the report*, which detailed the interview findings.

## Qualitative Results and Discussion

### Residents' Event Perspectives

Residents' event perspectives (i.e., perceptions, experiences, and insights) were discussed two-fold: in relation to micro (i.e., residents' individual perspectives from the event) and macro perspectives (i.e., the use of tax dollars). First, we elicited residents' perceptions to understand how individuals felt Canadian tax dollars were being used. On one hand, the 14 residents unanimously echoed that public funding was used to host the event (i.e., the actual act of hosting competitions, housing athletes, and fans) and build infrastructure throughout the province, such as the Sea-to-Sky Highway, which runs from Vancouver to Whistler (Sant and Mason, 2015; Van Wynsberghe, 2016). On the other hand, and again unanimously, residents did not discuss the use of Canadian tax dollars linked to other anticipated outcomes from the event, such as promoting Canadian industries abroad. In contrast, the documents analyzed indicated how Canadian tax dollars were used to promote Canadian industries domestically and abroad; to promote Indigenous communities, a key stakeholder in the Vancouver event; to foster social programs; to invest in arts and culture; and to invest in Canadian innovation and business. These public funding targeted outcomes were all confirmed through the document analysis conducted for this study (i.e., BC Auditor, 2002; VANOC, 2009, 2010).

Second, all residents discussed their event experiences beyond the link to public funding contributions. These discussions resulted in three themes: (1) *time*; (2) *outcomes*; and (3) *want vs. need*. For *time*, residents regularly discussed their perspectives of the event's value and how this value shifted over time from pre-event, to during the event, to post-event. These changes were not unidimensional, as individuals noted how perceived value may have been negative pre-event—some displayed by select residents voting no in the public referendum to host the 2010 event—then moved toward a positively perceived value post-event because of the positive experience they had during and after the Games.

*I was strongly against hosting the Games beforehand. I even voted no in the referendum, I was one of the only people I knew against it. It created quite the conversational piece at the dinner table and when we [my partner and I] were with friends/family… I really did enjoy it… it was a good use of money. If I could go back, I would vote yes now*. (Resident 5)

Conversely, the opposite pattern was also found, as some individuals moved from a positive value pre-event to a less positive value post-event. For instance, one individual discussed their strong support of the event by hosting international visitors; this individual repeatedly mentioned how enjoyable it was to meet new people, be an ambassador, and experience the event. Although the Games had an overtly positive impact for this resident leading up to and during the event, as the following quotation indicates, it quickly dropped off post-event: “*I don't think I walked around with my Canada hat and mittens on long after the Games”* (Resident 2).

For *outcomes*, residents' perspectives could be linked to positive and negative outcomes, as well as tangible and intangible outcomes. Although all participants reminisced on tangible and intangible outcomes (e.g., infrastructure and feelings of pride when Canada won its first gold medal), distinct nuances were found between their positive and negative memories. For example, almost all participants did not hesitate to discuss positive tangible outcomes like infrastructure (e.g., Richmond Oval and Sea-to-Sky Highway) or negative tangible outcomes like damage caused by riots and the economic costs that could burden them:

*The [Richmond] Oval is one of the best things to come from the Games. It's the pinnacle of Richmond and it still gives me goosebumps driving by and seeing it. It's beautiful inside and out. I actually have my name in there three times. It's a special place to a lot of people*. (Resident 8) *Unfortunately, we do have a reputation for damaging things. The Stanley Cup series was one occasion; the Olympics is another. The riots that happened…it's a shame that people feel the need to do that. That's not right and it's not what we want the world to see. It's not who we are as Vancouverites*. (Resident 3)

However, in discussing intangible outcomes, while participants could almost automatically discuss positive outcomes like the connection they felt to Vancouver, other residents, and Canada as a whole, or the sense of accomplishment and ambassadorship they felt from hosting, participants struggled to identify negative intangible impacts that occurred, demonstrating an overtly positive perception toward the event. For example, multiple individuals openly stated how they could not remember any heartache from hosting and explicitly said they did not remember any damage to the athletes or poor image to the event: “*Honestly, no. I don't remember anything bad happening. The event was a great time! There weren't any issues I remember… it's not like anyone was harmed. No harm to any athletes during the Games”* (Resident 2). Despite this residents' response, the reality of the 2010 OWG is that resident riots occurred during the opening days of the competition and an athlete, unfortunately, passed away during a pre-competition practice run (IOC, 2014).

The third theme was linked to the narrative of “the rich get richer, while the poor get poorer” and the concept of *want versus need*, which several participants voiced throughout the interviews. As one resident stated, “*the Olympics was a nice want and was not something we needed, but something we, and politicians, wanted—so that's what we got”* (Resident 9). Several residents mentioned how the event created a faster production of infrastructure projects in development, such as the Sea-to-Sky Highway and Canada Line. Linked to much urbanization literature (e.g., Essex and Chalkley, 1998), the OWG was viewed by residents as a catalyst to improving (much needed) transportation and infrastructure within the Metro Vancouver Regional District (Sant and Mason, 2015; Van Wynsberghe, 2016). Nonetheless, almost all participants recognized the concept of privilege and their position to have these discussions, conscious that not all individuals were afforded the same experiences, which further marginalized some. Although displacement was not a central result of the 2010 OWG (c.f. Sant and Mason, 2015; Van Wynsberghe, 2016), present issues in the city, like the economic polarization between the poverty of the downtown east area and the wealth of West Vancouver, were voiced by many participants. Specifically, several participants felt “*more could have been done”* (Resident 12) to alleviate the challenges residents in Vancouver's downtown east area face on a day-to-day basis. Moreover, summarized nicely by one participant, the Olympics, to some extent, distracted from these city issues, “*even though we have all the infrastructure… it just seems like this big, huge, waste of money. There's just so many other priorities. I don't see the return on it for everyone”* (Resident 9).

### Foregone Opportunities

In discussing the foregone opportunities residents faced from hosting the 2010 OWG, residents provided a broad list of examples. Upon analysis, this list was compartmentalized into seven individual themes: (1) economic; (2) environment; (3) sport events; (4) professional sport franchises; (5) education; (6) healthcare; and (7) low-income housing.

Aligned with the interview guide and the monetary value exploration of this line of inquiry, all individuals noted the tax dollars could be used for purely economic ventures elsewhere (e.g., paying national debt).

*We didn't need the event. Sure, it was nice to have, but that money could have been used elsewhere. Our debt for one thing, we could have used that money to reduce the debt and maybe reduce the need for the increase in taxes we've seen*. (Resident 14)

Moreover, individuals echoed the strong environmental focus of the event and felt a possible way to use public funding would be to improve the city's (and country's) ecological footprint. Thus, rather than hosting a large event that comes with environmental challenges (Preuß, 2007; Collins et al., 2009), government officials could have used residents' public funding investment purely toward improving the ecological footprint.

*Listen, it's [British Columbia]. We are supposed to be the green capital of the country and hosting a major event like this has to come with some environmental…issues. I'm not saying it was a bad idea, but there are things we seem to care a lot about, environmental things, that don't align with this spending*. (Resident 4)

In addition, several sport options were suggested by the resident interviewees. This was done in one of two ways, either by having multiple smaller events—“*it would be nice to take the money and use it for events all over [British Columbia], or even Canada. Rather than one big event just for Vancouverites”* said Resident 7—or by bringing back beloved professional sport teams (e.g., Vancouver Grizzlies, NBA Franchise based in Vancouver until 2001; Chiba, 2012).

*If we're choosing how to spend money on sport, sport teams would be great, not just youth options but bringing back pro teams. The city was a buzz during the [Toronto] Raptor's playoff run, it would be really fun having the [Vancouver] Grizzlies' back*. (Resident 7).

Moreover, residents suggested both education (e.g., greater public-school funding) and healthcare opportunities (e.g., build more hospitals) could be alternative options for their taxpayer contributions, often linked to the present need for education and health surrounding the COVID-19 Pandemic during the time of these interviews. While multiple interviewees discussed these foregone opportunities at large, perhaps the forgone opportunity voiced most loudly was linked to Vancouver's downtown east side and the need for low-income housing.

*It's a need we've always had and it's a need we still have. We struggle every day and it's hard to think about all that money and how it went toward hosting an event rather than helping Vancouverites in need*. (Resident 12).

These seven foregone opportunities mentioned by the interviewees align with those identified by sport management scholars (e.g., Preuß, 2009; Karadakis et al., 2010; Taks et al., 2011) who proposed alternative options encompassing various economic, tourism, environmental, social, cultural, psychological, and political foregone opportunities.

These findings highlight the temporal nature of residents' major sport event perspectives and the reality of host city residents' post-event perspectives. While residents revealed positive event perspectives, like previous research on residents' referendum perceptions (Scheu and Preusß, 2018; i.e., Johnston et al., 2021b,c), interviewees voiced negative event legacy perspectives and concerns around using public funding to host the major sport event. Consequently, these findings not only revealed the complex entanglement of positive and negative perspectives scholars know host city residents hold (e.g., Karadakis and Kaplanidou, 2012; Johnston et al., 2021a) but revealed that positively perceived events may still lack a positive social value for such residents.

This alarming finding not only further advances the need to answer this study's overall purpose but also challenges the practical applicability of previous sport management research, which has exclusively focused on residents' intangible perceptions (e.g., Bakhsh et al., 2018; Oja et al., 2018; Park et al., 2019) or experiences (e.g., Taks et al., 2020; Oshimi et al., 2021) and not on a more tangible monetary valuation (Preuß and Hong, 2021). Based on our findings, as positive event perspectives do not necessarily indicate positive event valuations, more tangible valuations should be conducted on major sport event host city residents. Ultimately, these interviewee responses and document analysis informed the questionnaire's development (discussed in the following section) and anticipated findings from the *reverse contingent valuation method* metrics and *opportunity cost approach* selections.

## Quantitative Method

### Quantitative Data Collection

Using the *Qualtrics* platform, Vancouver residents were invited to participate in an online self-administered questionnaire. The research team received ethics approval from their university ethics board (#XXX) for this step. Qualtrics first screened all participants to ensure they knew the City of Vancouver hosted the 2010 OWG, born in 1984 or earlier, and were residents of the City of Vancouver. Like the interview selection criteria, this ensured all participants were of legal voting age during the referendum and could reflect on their hosting experience as a Vancouver resident.

The questionnaire was distributed on February 15, 2021, with all participants completing the questionnaire by February 24, 2021. Using statistical power analysis with confidence levels at 95%, a margin of error at 5%, and a 610,000 population size for the City of Vancouver, a minimum sample size of 385 randomly selected residents was needed (*n* = 385; Cohen, 1988). To ensure this minimum threshold, a quota sample of 525 Vancouver residents was targeted and collected through Qualtrics panel data members who identified as being Vancouver residents and born in 1984 or earlier, with no respondents having previously participated in the study's interviews. All Qualtrics panel members had the choice to participate or not participate in the study. At no point did the research team have access to contact participants directly, the questionnaire was built on the Qualtrics platform and then the Qualtrics team disseminated the questionnaire to these participants and provided the raw data set to the research team.

The sample showed an even divide between genders; 52.6% identified as female (*n* = 276) and 46.7% identified as male (*n* = 245), while four individuals identified as non-cis (0.8%). Participants were born between 1935 and 1984, with an average birth year of 1963.83 (*SD* = 12.14). Most participants identified as Caucasian (*n* = 291; 55.4%) with a high proportion of participants identifying as Chinese (*n* = 113; 21.5%). Most participants also identified as having completed either a university bachelor's degree or college diploma (*n* = 268; 51.0%) and being employed to some degree (e.g., full-time, part-time, self-employed; *n* = 348; 66.3%) with the remaining 33.7% of participants identified being unemployed, retired, on a government assistance program, or a full-time student. In addition, respondents' average income was $76,700 CAD (*SD* = $55,560). Finally, an important step when using panel data, these socio-demographic variables were confirmed with Canadian census data on the City of Vancouver by showing linearity across all metrics except income, where study participants revealed an average income ~$15,000 higher than the Census report (Statistics Canada, 2017). This is important to understand upfront, as this higher average income may be an underpinning factor in creating a positive social value for residents.

### Questionnaire Scenarios and Measures

#### Reverse Contingent Valuation Method

Two elements are required to apply a *reverse contingent valuation method* (Humphreys et al., 2018; Orlowski and Wicker, 2019): a scenario and an associated evaluation. The scenario informs respondents of the necessary information needed to make their valuation (e.g., impacts of the event, event investment). The scenario built for this questionnaire was informed from Vancouver residents' perspectives understood in the previous qualitative phase and supplemented by the insights learned from the document analysis. The evaluation was asked in a two-part fashion, as determining a *reverse contingent valuation method* metric is a two-part consumption decision. In decision one, the participant was asked if they would use (or not) public funding to host the 2010 OWG. Following this scenario, participants were provided the options of *yes* (i.e., they do support having used tax dollars to host the 2010 OWG) or *no* (i.e., they do not). Finally, those who answered yes and indicated support to the first decision were asked how much they would be willing to provide to host the 2010 OWG (i.e., decision two).

For this second decision response, respondents answered on a scale ranging from $1 to $250, where individuals could select any whole dollar figure (e.g., $1, $2, $3). This scale was chosen for two reasons. First, aligned with appropriate *reverse contingent valuation method* measures, the lowest option for the consumption decision must be offered to the respondents. Since the initial decision asked if individuals would support using tax dollars, not a specific amount of tax dollars, the minimum assumed must be $1. Second, through the previous document analysis, Vancouver's taxpayers estimated actual tax dollars would have been between $75 and $175 per year. With the middle point of this estimation being $125, that point was used as the middle point of the scale, creating a $250 extremity. Refer to Appendix B for the *reverse contingent valuation method* survey item.

#### Opportunity Cost Approach

To apply an opportunity cost approach monetary valuation method, respondents must be presented with a sound depiction of the non-market value they received (e.g., hosting the 2010 OWG) and opportunities foregone. Informed from the resident interviews in the previous data phase, survey participants were presented with a scenario detailing the taxpayer contributions, event impacts, and foregone opportunities. Following this scenario, participants were provided the options of *yes* (i.e., they do support having used $2b to $4b of Canadian tax dollars to host the 2010 Games) or *no* (i.e., they do not).

First, for those who said *no* to the initial item, they were provided with the seven alternative options suggested by the results from the previous phase: (1) decrease national debt; (2) improve Canada's ecological footprint; (3) fund other major sport events; (4) bring back Canadian professional sport teams; (5) increase Canada's post-secondary education opportunities; (6) build more hospitals and medical buildings; and (7) develop low-income housing. In addition to these seven options, which are also endorsed by previous studies, an eighth option was offered (i.e., other), where responds could enter their own alternative if the list of seven options did not represent all alternatives they deemed important.

From these eight options, participants were asked to rank their top three choices in order of what they would select as better alternatives to allocate that funding than for hosting the 2010 OWG. This data is reported in Table 2 and outlines the eight options based on the percentage of *non-supporting residents* (*n* = 184: those who said no to the initial *opportunity cost approach* item) who selected each option in their top three preferences. For example, *hospitals* (69.0%) indicates that 69.0% of the *non-supporting residents* (*n* = 127) selected *hospitals* as one of their top three alternatives to allocate better the public funding than hosting the 2010 OWG.

**Table 2:** Micro-Level social value perspective: reverse contingent valuation method.

	**Total sample**	**Willingness to pay sample**
	**(*n* = 525)**	**(*n* = 409)**
	**%**	**%**
Not willing to pay	22.1	–
Willing to pay < $75	36.4	46.7
Willing to pay $75–175	33.9	43.5
Willing to pay >$175	7.6	9.8

Second, all individuals – regardless of responding yes or no to the initial *opportunity cost approach* item—were presented a new *opportunity cost approach* item which first depicted their estimated taxpayer contributions (i.e., $75 to $175 each year for 7 years, in present value), and then offered the same eight options with the addition of still funding the 2010 OWG as a ninth option. Refer to Appendix C for the *opportunity cost approach* scenario. This second *opportunity cost approach* evaluation allowed us to examine residents' hosting preferences in relation to other preferences (i.e., alternative options), which was done for both *non-supporting residents* (*n* = 184: those who said no to the initial *opportunity cost approach* item) and *supporting residents* (*n* = 341: those who said yes to the initial *opportunity cost approach* item). In doing so, the same preference percentage findings outlined above for *non-supporting residents* are reported in Table 2 for *supporting residents*. For example, *Olympics* (75.7%) indicates that 75.7% of the *supporting residents* (*n* = 285) selected *Olympics* as one of their top three choices for the public funding used to host the 2010 OWG. Moreover, the preference percentage findings for *non-supporting residents* are also outlined in Table 2 when the additional option of hosting the OWG was included (i.e., with the *Olympic* option). This sequence allowed us to check the congruency and changes of *non-supporting residents'* top three funding choices when presented and not presented the option to host the 2010 OWG.

## Quantitative Results and Discussion

### Micro-Level Perspective: Reverse Contingent Valuation Method

Of the 525 Vancouver residents who completed the questionnaire, the willingness-to-pay metric (see Appendix B) indicated most respondents supported using public funding to host the 2010 OWG (77.9%, *n* = 409). Of these 409 individuals, the average willingness-to-pay metric was $86.39 (*SD* = $62.19) each year for the seven years, establishing the average total contribution individuals were willing to pay to host the 2010 OWG as $604.73. These contribution findings reveal four distinct groups linked to the estimated taxpayer contributions outlined in the *opportunity cost approach* item. First, 22.1% (*n* = 116) individuals indicated they would not be willing to pay to host the 2010 OWG. Second, 36.4% (*n* = 191) individuals indicated they would be willing to pay < $75 each year, which falls short of the estimated taxpayer contribution of $75 to $175 Vancouver residents made. Third, 33.9% (*n* = 178) individuals indicated they would be willing to pay between $75 and $175 each year, which falls within the estimated parameters of Vancouver residents' actual taxpayer contributions. Finally, the remaining 7.6% (*n* = 40) individuals indicated they would be willing to pay more than their estimated taxpayer contributions (i.e., more than $175 a year). These initial findings suggest that, while most respondents were willing to pay to host the 2010 OWG (i.e., 77.7%, *n* = 409), what they were willing to invest *post-hoc* did not exceed that investment. See Table 2 for percentage breakdowns of the total sample and willingness-to-pay sample.

These initial findings indicate that Vancouver residents' social value does not equate the actual monetary value contributed. Thus, hosting the 2010 OWG may not have been a positive investment for Vancouver residents and offers support to previous scholarship which suggests hosting major sport events may not provide the best value for money, namely a high enough positive social value for host city residents (e.g., Barros, 2006). In total, 485 (92.4%) of respondents indicated they would not be willing to pay more than the maximum estimated contribution of $175. This finding challenges the claimed belief that hosting major sport events will bring positive social experiences which outweigh their financial costs (Doyle et al., 2021). Moreover, these findings reveal that a post-event willingness-to-pay method (or *reverse contingent valuation method*) is a suitable and practical tool to directly elicit residents' monetary value of major sport event social perspectives. Ultimately, applying this reverse contingent valuation method revealed the usefulness of this method to garner a micro-level perspective of residents' social value and indicated that Vancouverites supported the notion of publicly funding the 2010 OWG, but the social value they garnered from that hosting experience did not match their estimated taxpayer contribution.

### Macro-Level Perspective: Opportunity Cost Approach

When presented with the initial *opportunity cost approach* item (refer to Appendix C), 65.0% of Vancouver residents (*n* = 341) indicated they would support using the $75–$175 yearly taxpayers' contributions for 7 years as public funding to host the 2010 OWG. However, of the individuals who did not support using this public funding to host the event (*n* = 184), the most popular alternatives respondents ranked in their top three choices were *hospitals* (69.0%), *national debt* (65.2%), and *housing* (57.6%). Next, when asked to rank the same options with the additional choice of still funding the 2010 OWG, these 184 individuals' responses showed consistency, as *hospitals* (60.9%), *national debt* (60.3%), and *housing* (51.6%) maintained their status as the top three selections. However, in this particular group, the *Olympic* option did now appear for 44 respondents who put it in their top three choice selections (23.9% of individuals). This suggests that, while 184 respondents did not support using public funding to host the Games initially when provided with alternative options, it is possible they felt hosting the Olympics was as good an alternative option as the others offered. This finding supports and magnifies much of the tension residents indicate around hosting publicly funded major sport events: while both positive and negative perspectives exist (Bakhsh et al., 2018; Johnston et al., 2021a), residents are often left uncertain of what option to support (Johnston et al., 2021b).

Conversely, for the 341 individuals who did support using public funding to host the 2010 OWG, the *Olympics* (75.7%) was the overwhelming top three selection by respondents, with *national debt* (51.0%) and *environment* (45.2%) rounding out the top three selections. Moreover, to further amplify this top three preference finding, the *Olympics* received 173 first selection votes (50.7%). In other words, most respondents who supported using the estimated public funding to host the 2010 OWG still selected to use that funding to host the event over alternative options like increasing hospitals and medical buildings, improving the ecological footprint, and decreasing national debt. Table 3 presents the complete alternative rankings provided for residents who did and did not support using public funding to host the 2010 OWG.

**Table 3:** Macro-Level social value perspective: opportunity cost approach.

	**Non-supporting residents** **(*****n*** **= 184)**				**Supporting residents** **(*****n*** **= 341)**	
	**Without Olympic option**		**With Olympic option**		**With Olympic option**	
**Ranking**		**%**		**%**		**%**
1	Hospitals	69.0	Hospitals	60.9	Olympics	75.7
2	National debt	65.2	National debt	60.3	National debt	51.0
3	Housing	57.6	Housing	51.6	Environment	45.2
4	Environment	46.7	Environment	47.8	Hospitals	42.8
5	Education	39.7	Education	29.9	Housing	42.5
6	Other	9.2	Olympics	29.3	Education	23.5
7	Sport events	7.6	Other	10.9	Sport events	10.3
8	Pro sport teams	4.9	Sport events	6.0	Pro sport teams	5.6
9	–	–	Pro sport teams	3.3	Other	3.5

*Respondents' main options for “other” included tax reductions, infrastructure, and poverty/homelessness. Percentage indicates the number of respondents who placed the option as one of their top three selections*.

Unlike the tensions revealed for the *non-supporting residents*, those who supported hosting the 2010 OWG from the outset of the *opportunity cost approach* item (i.e., *supporting residents*) did not waver in their decision when presented alternative options. However, when comparing macro and micro perspectives, the disparity is revealed. From a macro perspective, 173 individuals (33.0% of the total sample) selected the *Olympic* as their first choice in the *opportunity cost approach* item after being presented their estimated taxpayer contributions (i.e., $75–$175 each year for 7 years). From a micro perspective, 191 individuals (36.4% of the total sample) revealed they would willingly contribute the same amount or more (>$75 each year for 7 years) in the *reverse contingent valuation method* item. Although close, this change suggests that hosting a major sport event may receive less support when residents are presented with alternative options or more information about the choices at hand (Johnston et al., 2021b). This finding reveals the importance of providing transparency to help residents make informed decisions regarding the use of their taxpayer contributions (Davies et al., 2019, 2021); it also illustrates that the way in which a survey is framed affects the results (Lumsdaine and Exterkate, 2013). Ultimately, applying this opportunity cost approach revealed the usefulness of this method to garner a macro-level perspective of residents' social value.

## Overall Discussion

This study sought to investigate various monetary valuation methods that could be used to analyze residents' social value in a post-event context and tested the selected methods to determine residents' social value form hosting the 2010 OWG. First, our qualitative findings revealed host city residents' positive hosting perspectives and highlighted their concerns of a positive monetary event valuation (i.e., positive social value). This finding was then confirmed through both monetary valuation methods used in the quantitative part of the study. The *reverse contingent valuation method* indicated most individuals attributed a lower monetary value to their social experiences and insights 11 years post-event compared to their estimated taxpayer contribution; the *opportunity cost approach* revealed fewer compared to their monetary investments and challenge whether this event in particular left legacies worth the investment and if these public funds were efficiently used (Barros, 2006; Scheu and Preusß, 2018).

Second, after conducting this mixed methods study, our findings revealed that both monetary valuation methods have benefits, and their complementary micro and macro-level perspectives can be advantageous. Specifically, as both methods have benefits and challenges to measuring social value, combining these methods may offer a valuable tool to measure the social value of a major sport event. From a conceptual perspective, this synergy offers a holistic approach, whereby the micro-level perspective serves as a prerequisite to make a better-informed decision, or generate a better-informed macro-level perspective, thereby more accurately estimating residents' social value. On the one hand, the *reverse contingent valuation method* provided a direct measure of residents' social value at the individual level (i.e., micro perspective; Humphreys et al., 2018), while on the other hand, the *opportunity cost approach* helped frame that social value amongst other valued opportunities/scenarios at the societal level (i.e., macro perspective; Salamon et al., 2011; Orlowski and Wicker, 2015). From an empirical view, the micro perspective allowed for a tangible monetary value compared to residents' actual taxpayer contributions, while the macro perspective adds depth to the analysis by further explaining the value of hosting in relation to other contextual ventures (e.g., decrease national debt, improve ecological footprint; Preuß et al., 2007).

The combination of these methods thus provides an answer to which tool(s) to use and provides insight into the unique contribution of each monetary valuation method to elicit social value. Although both methods have their respective merits when measuring the social value of a major sport event (Orlowski and Wicker, 2019), the findings from this study indicate the *opportunity cost approach* (i.e., macro perspective) is a complementary addition to the necessary micro perspective offered by the *reverse contingent valuation method*. Consequently, we first discuss the merits of the individual methods from this study and then conclude with how the two methods can best be used together to measure social value.

### Micro-Level Perspective: Reverse Contingent Valuation Method

As a stated preference monetary valuation method (Orlowski and Wicker, 2019), the *reverse contingent valuation method* allowed us to determine residents' value of hosting the 2010 OWG after having already experienced the referendum, pre-event build-up, the event itself, and immediate 11 years post-event. In doing so, this method provides the key to determining social value for individuals—the monetary value of a social experience (Humphreys et al., 2018; Preuß and Hong, 2021). By using this method, we can evaluate residents' monetary value of their hosting experience as a direct individual-level measure, and this measure can then be compared to residents' estimated taxpayer contributions to determine how the perceived social value compares to the monetary investment (e.g., positive, negative, or equal).

Although this method provides the necessary tool to measuring social value (i.e., direct measure), discussing and selecting social value numbers for large amounts (e.g., overall event funding) is still a challenge for two reasons. First, macro valuations of major sport events are often extreme monetary values residents are disconnected from in their daily lives (Keane et al., 2019). Because of this disparity, it is helpful to examine the individual level first so that residents can gauge a better understanding of the larger (i.e., macro) picture. Second, from a contextual perspective, the tangibility of a billion-dollar valuation is out of reach for most individuals and may perpetuate an intangible understanding. In this study, we attempted to mitigate this challenge by scaling the valuation for residents to elicit their individual contributions (i.e., micro perspective) instead of only fixating on the overall societal contribution (i.e., macro perspective). Nonetheless, when used on its own, the *reverse contingent valuation method* lacks contextual depth, and although it produces a direct measure at the individual level, it is challenged to measure social value at the societal/macro level.

### Macro-Level Perspective: Opportunity Cost Approach

As a revealed preference monetary valuation method (Orlowski and Wicker, 2019), the *opportunity cost approach* allowed us to examine the priority of residents' alternative options to hosting the 2010 OWG without needing the monetary value of those options. Instead, by having the monetary value of taxpayer contributions to host, we could use this method to determine which alternatives (e.g., decrease national debt, build more hospitals) were or were not a higher priority to residents. As discussing macro-level perspectives (e.g., overall event funding) can be challenging for individuals, offering priority choices through the *opportunity cost approach*, while not a direct measure, can provide great depth to understanding individuals' perspectives of the social value of sport events at the society level.

However, the *opportunity cost approach* alone does not offer a solution to measuring social value. Although it provides contextual understandings and elicits preferences, it is bound by the alternative options presented (Orlowski and Wicker, 2019). Consequently, it cannot offer a social value measure beyond simply stating if it is or is not a greater value than a select forgone alternative option. In the present study, because all alternative options were intangible to some degree (e.g., bring back professional sport teams), the *opportunity cost approach* could not offer a tangible measure needed to determine a major sport event's social value.

### Hybrid Method

Our findings indicate benefits from examining both residents' micro- and macro-level perspectives. Social value is a complex concept, one ripe with entangled aspects and perspectives (Ziakis, 2016; Davies et al., 2021; Preuß and Hong, 2021). Consequently, solely determining micro (e.g., individual's willingness-to-pay) or macro perspectives (e.g., foregone opportunities) may not always provide the in-depth analysis needed to match the complexities of social value. Depending on the context of the study, combining both may be most fruitful for researchers and practitioners. Demonstrated in this study, there were benefits to combining the micro and macro perspectives. On the one hand, the micro perspective provided us the ability to make social value evaluations at the individual level and help individuals generate a more informed opinion of an event's social value at the societal level. On the other hand, the macro perspective provided a depth of understanding which moved the evaluation from a mere number to an understanding of residents' perspective of the event's social value at the societal level.

Our support for this hybrid method does not discount the importance of each method (i.e., *reverse contingent valuation method* and *opportunity cost approach*) being examined individually. Although this study benefited from including both the micro and macro perspectives, this may not be the best option for all future studies. It is clear from these findings that there is a benefit to conducting studies using each of the individual methods, specifically based on which level is most important for the research purpose. For some studies, this may be a micro only perspective, a macro only perspective, or a micro and macro perspective. Much of this decision may be central to the data researchers have available to them. For instance, in the present study, although data were available for both the micro and macro level perspectives, the estimated taxpayer contributions and individual experiences examined at the micro level appeared more tangible than the estimated taxpayer contributions and hypothetical alternatives to hosting at the macro level. Understanding that the micro level data was more tangible than the macro level data for this study may be largely why the micro perspective was the key driver of examining residents' social value while the macro perspective was an additional layer of knowledge. Consequently, although the hybrid method used in this study to examine residents' social value was advantageous, it is clear that the micro perspective examination was more relevant in the context of residents' social value. This does not mean micro perspectives are more important than macro, nor that this hybrid method should always be used moving forward. Rather, it is necessary to examine residents' social value aligned with the perspectives of the participants, the event context, and based on the data available.

## Implications

Regarding theoretical implications, researchers should consider both micro and macro perspectives when examining major sport events' social value. Aligned with the preferences and exchanges individuals and groups endure when hosting a publicly-funded major sport event, this study highlights how eliciting a micro and macro perspective to measure residents' major sport event social value can be advantageous. Scholars interested in examining the social value of major sport events need to understand the complexity of public funding, anticipated outcomes, stakeholder perspectives, and micro and macro social value evaluations. Consequently, they should justify why a micro or macro only perspective is beneficial or why a hybrid method is most fruitful for the study in question. Our findings highlighted the benefit of adding the macro perspective to the micro perspective which allowed us to understand residents' social value at greater depths. This deeper focus on the how and why aspects of social value will allow us as researchers to better address stakeholder claims, accurately measure social value, and ultimately improve the social value generated by major sport events.

Regarding practical implications, this study offers practitioners a tool to monetarily value residents' social experiences from hosting a major sport event. Determining such social value alone can allow practitioners to make evidence-based decisions circled around public funding in one of two ways. On one hand, like the post-event evaluation conducted in this study, practitioners can evaluate residents' experience in relation to their actual taxpayer contributions. On the other hand, practitioners can use this hybrid-type method pre-event or during the bid phase to determine what amount of public funding would or would not be an efficient and supported use by residents. However, although these benefits are certainly present, understanding a major sport event's social value is in its infancy. Thus, practitioners should be cautioned that we have only scratched the surface of understanding these numbers.

## Study Limitations and Future Research Directions

First, methodological challenges were present when attempting to garner accurate public funding estimations. As these are necessary metrics for conducting social value studies, we encourage scholars and practitioners to triangulate their data through event experts, official financial and event documents, and official event auditing documents, as we have done in this study. This combination proved fruitful in garnering estimating realistic numbers and developing a greater understanding of the overall event funding.

Second, examining social value 11 years post-event means there may be memory reliance challenges and experiential biases. As our interviews demonstrated, the inability to recall negative intangible outcomes was present. This limitation is not necessarily one that researchers and practitioners can correct. Rather, it is an important underpinning reality scholars and practitioners must be conscious of when conducting extensive post-event evaluations.

Third, the residents' selected for this study were only those of the Vancouver region (i.e., host region). However, residents internal and external to the Vancouver region were affected socially and economically from hosting the 2010 OWG (Karadakis and Kaplanidou, 2012; Sant and Mason, 2015; Van Wynsberghe, 2016; Humphreys et al., 2018). Although investigating Vancouver residents' social value, this study does not provide insight into other important (and investing) residents (e.g., residents in cities like Whistler and Richmond which hosted official OWG events). Given that federal public funding was used to host the event and all Canadians were impacted from hosting, future scholars should aim to conduct social value studies which encompass all affected residents or resident groups involved in this socio-economic exchange.

Finally, although much scholarship is centralized around the positive perception of hosting major sport events, our findings indicate positive perceptions do not necessarily result in a social value that outweighs the financial investment (i.e., taxpayer dollars). However, with the identification of this hybrid monetary valuation method, researchers and practitioners are now equipped with the tools to examine and understand the social value major sport events can create. Thus, we encourage all stakeholders to shift away from perception-centric conversations, apply appropriate monetary valuation methods, and move toward examining the social value of hosting major sport events through residents' perspectives at the individual (i.e., micro) and societal (i.e., macro) levels.

## Data Availability Statement

The raw data supporting the conclusions of this article will be made available by the authors, without undue reservation.

## Ethics Statement

The studies involving human participants were reviewed and approved by University of Ottawa Research Ethics Board. The patients/participants provided their written informed consent to participate in this study.

## Author Contributions

All authors listed have made a substantial, direct, and intellectual contribution to the work and approved it for publication.

## Conflict of Interest

The authors declare that the research was conducted in the absence of any commercial or financial relationships that could be construed as a potential conflict of interest.

## Publisher's Note

All claims expressed in this article are solely those of the authors and do not necessarily represent those of their affiliated organizations, or those of the publisher, the editors and the reviewers. Any product that may be evaluated in this article, or claim that may be made by its manufacturer, is not guaranteed or endorsed by the publisher.

## Footnotes

^1^All monetary values presented in this study are in Canadian currency (i.e., CAD).
