# Supplementary material for: Examining Monetary Valuation Methods to Analyze Residents' Social Value From Hosting a Publicly-Funded Major Sport Event
Source: Front Sports Act Living. 2022 Mar 23;4:823191. doi: 10.3389/fspor.2022.823191 (PMC8983964; doi:10.3389/fspor.2022.823191)
Supplement: Supplementary file 1 [file Data_Sheet_1.docx]

**Appendix**

**Appendix A – Semi-Structured Interview Guide**

**Introduction**

Thank you for participating in this study. Your involvement is greatly appreciated and will provide valuable insights for my research project.

Before we start, can you confirm you were born before 1984?

🡪 if yes, continue

🡪 if no, thank the participant for his/her willingness to participate

Can you confirm, you are a resident of [City]

🡪 if yes, continue

🡪 if no, thank the participant for their willingness to participate

**Demographic Information**

- Where do you currently live?
- Where did you live at the time of the referendum (February 2003)?
- Where did you live while the 2010 Games were being planned (between July 2003 and December 2009)
- Where did you live at the time of the 2010 Games (February 2010)?
- How old were you at the time of the referendum?
- Did you vote in the referendum? If yes, can recall your vote? If no, why did you choose not to vote at the referendum?
- Can you tell me about your experience with the referendum (e.g., was it something you would talk about with others; did you follow it in the media)?
- Can you tell me about your experience leading up to hosting the Games (e.g., was it something you would talk about with others; did you follow it in the media)?

**Legacies**

1. As a **[________]** resident, what expectations did you have for the City of Vancouver from hosting the 2010 Games?
   - Were they positive? Negative?
   - Why did you have these expectations?
     - Community needs?
     - Resident needs?
2. Were any of these expectations fulfilled?
   - Which ones were?
     - Why do you think these expectations were fulfilled?
     - Are you able to place a timeline on when you feel this/these occurred?
   - Which ones were not?
     - Why do you think these expectations have not been fulfilled yet?
     - Will/can these expectations ever be fulfilled?
3. Why do you think Vancouver wanted to host the 2010 Olympic Winter Games?
   - Community
   - Economy
   - Political
   - Environmental
   - Infrastructure
   - Sport Participation
4. Do you feel that hosting the 2010 Games affected/changed your life personally or your life in the community, or the community at large? Please explain or give me some examples.

**Return on Investment**

1. How important was hosting the 2010 Games event to you?
   - Did Vancouver need the Games?
   - Was it the right time to host?
   - Do you think the Games brought about change?
     - Positive change? Negative change? Needed change?
2. Do you think hosting the Games was a good use of public funding?
   - Why or why not?
   - How else could the money have been spent?
3. Looking back, was hosting the Games a good idea?
   - Would you host again?
     - Why or why not?
   - Should other Canadian communities host?
     - Why or why not?
4. Can you tell me a positive story from hosting the 2010 Games?
5. Can you tell me a negative story from hosting the 2010 Games?

Is there anything else you would like to mention regarding hosting the 2010 Games and its legacies?

**Appendix B – Reverse Contingent Valuation Method Item**

The following scenario and question ask about your willingness to pay for the **2010 Games** having experienced the event more than 10 years ago.

To host the **2010 Games** in the City of Vancouver, a large portion of funding was provided from Canadian, British Columbian, and Vancouver tax dollars. Some of these tax dollars were used specifically for hosting the event, while other tax dollars were used to promote Canada domestically and abroad, foster social programs, promote Indigenous communities, invest in arts and culture, and build the Sea-to-Sky Highway (highway from Vancouver to Whistler).

In addition, tax dollars were used to build infrastructure in British Columbia like improving the Sea-to-Sky Highway (the highway from Vancouver to Whistler) and accelerated construction of the Vancouver Convention Centre and Canada Line (infrastructure in British Columbia).

Many Canadians have voiced how the 2010 Games brought positive and negative social impacts to their lives, like community disruption, national pride, community displacement, and sport participation opportunities.

Now, more than 10 years later, as a Canadian who experienced this event, do you support having used tax dollars to host the **2010 Games**?

- Yes
- No

**For those who answered “Yes”:**

Canadians’ tax dollars were used each year for the 7 years leading up to the **2010 Games**. Considering your current income, how much of your current tax dollars (in present value) would you support having been used to host the **2010 Games** each year for 7 years?

For example, if you say $10 on the following scale, that is $10 a year for 7 years, a $70 total; if you say $100 a year for 7 years, that is a $700 total.

$25 $50 $75 $100 $125 $150 $175 $200 $225

$1 ---------I---------I--------I--------I--------I--------I--------I---------I--------I----- >$250

**Appendix C – Opportunity Cost Approach**

**Q3.c.** An estimated **$2 to $4 billion (in present value)** of Canadians’ taxes were spent over seven years to host the **2010 Games.** First, **$2 billion (in present value)** was used to host the event, promote Canada domestically and abroad, foster social programs, promote Indigenous communities, invest in arts and culture, and build the Sea-to-Sky Highway.

In addition, **$2 billion (in present value)** were used to develop the Vancouver Convention Centre and Canada Line (infrastructure already in development but fast-tracked to be ready for the **2010 Games**).

In total, this creates a **$2-4 billion (in present value)** estimated public investment, over a 7 year period to host the **2010 Games.** It is possible some of this money could have been spent elsewhere (e.g., decrease national debt, improve Canada’s ecological footprint, develop low-income housing).

If Canada let another country host the 2010 Games, Canadians would still have been able to follow the event via television broadcast and used some of these tax dollars elsewhere. Also, infrastructure like the Vancouver Convention Centre, Sea-to-Sky Highway, and Canada Line in British Columbia may not have been completed or may have taken much longer to complete.

Now, more than 10 years later, as a person who experienced this event in your community, do you think it is okay **$2 to 4b billion (present value)** of tax dollars were used over seven years to host the **2010 Games**?

- Yes
- No

**For those who answered “No”:**

From the following list, please rank your top 3 options in order of how you would feel this money could have been better used for instead of hosting the 2010 Games. (Rank your top 3 by dragging the options into your desired order).

You can also use the “other” option to identify additional alternatives for spending these tax dollars.

___ I would decrease national debt

___ I would improve Canada’s ecological footprint

___ I would increase Canada’s post-secondary education opportunities

___ I would fund other major sport events in Canada (e.g., Commonwealth Games)

___ I would bring back Canadian professional sport teams (e.g., Vancouver Grizzlies,

Québec Nordiques)

___ I would develop low-income housing in Canada (e.g., downtown east Vancouver,

downtown central Winnipeg)

___ I would build more hospitals and medical buildings in Canada

___ Other __________________________

**For all participants:**

The following is an estimated average for what each taxpaying Canadian would have paid in present (2021) value to host the **2010 Games**.

Vancouver resident $75-$175 per year, for 7 years ($525-$1225 total)

British Columbia resident (outside of Vancouver) $65-$130 per year, for 7 years ($455-$910 total)

Canadian resident (outside of British Columbia) $4-$7 per year, for 7 years ($28-$49 total)

Now that you know how much the 2010 Games approximately cost you, please rank your top 3 options in order of how you would allocate the overall **$2 to $4 billion** **(present value)** in tax dollars. (Rank your top 3 by dragging the options into your desired order).

You can also use the “other” option to identify additional alternatives for spending these tax dollars.

___ I would still provide the funding to host the 2010 Games

___ I would decrease national debt

___ I would improve Canada’s ecological footprint

___ I would increase Canada’s post-secondary education opportunities

___ I would fund other major sport events in Canada (e.g., Commonwealth Games)

___ I would bring back Canadian professional sport teams (e.g., Vancouver Grizzlies,

Québec Nordiques)

___ I would develop low-income housing in Canada (e.g., downtown east Vancouver,

downtown central Winnipeg)

___ I would build more hospitals and medical buildings in Canada

___ Other __________________________
